# Lymphodepletion – an essential but undervalued part of the chimeric antigen receptor T-cell therapy cycle

**DOI:** 10.3389/fimmu.2023.1303935

**Published:** 2023-12-22

**Authors:** Benno Lickefett, Lulu Chu, Valentin Ortiz-Maldonado, Linda Warmuth, Pere Barba, Matteo Doglio, David Henderson, Michael Hudecek, Andreas Kremer, Janet Markman, Magdalena Nauerth, Helene Negre, Carmen Sanges, Philipp B. Staber, Rebecca Tanzi, Julio Delgado, Dirk H. Busch, Jürgen Kuball, Maik Luu, Ulrich Jäger

**Affiliations:** ^1^ Department of Medicine I, Division of Hematology and Hemostaseology, Medical University of Vienna, Vienna, Austria; ^2^ Cell Therapy Clinical Pharmacology and Modeling, Takeda, Boston, MA, United States; ^3^ Department of Hematology, Hospital Clínic de Barcelona, Barcelona, Spain; ^4^ Institut für Med. Mikrobiologie, Immunologie und Hygiene, Technische Universität Munich, Munich, Germany; ^5^ Hematology Department, Hospital Universitari Vall d’Hebron, Barcelona, Spain; ^6^ Experimental Hematology Unit, IRCCS San Raffaele Scientific Institute, Milano, Italy; ^7^ Bayer Aktiengesellschaft (AG), Business Development & Licensing & Open Innovation (OI), Pharmaceuticals, Berlin, Germany; ^8^ Lehrstuhl für Zelluläre Immuntherapie, Medizinische Klinik und Poliklinik II, Universitätsklinikum Würzburg, Würzburg, Germany; ^9^ ITTM S.A. (Information Technology for Translational Medicine), Esch-sur-Alzette, Luxembourg; ^10^ Institut de Recherches Internationales Servier, Suresnes, France; ^11^ Legal and Regulatory Affairs Committee of the European Society for Blood and Marrow Transplantation, Leiden, Netherlands

**Keywords:** CAR-T cells, lymphodepletion, conditioning, optimization, efficacy, toxicity

## Abstract

Lymphodepletion (LD) or conditioning is an essential step in the application of currently used autologous and allogeneic chimeric antigen receptor T-cell (CAR-T) therapies as it maximizes engraftment, efficacy and long-term survival of CAR-T. Its main modes of action are the depletion and modulation of endogenous lymphocytes, conditioning of the microenvironment for improved CAR-T expansion and persistence, and reduction of tumor load. However, most LD regimens provide a broad and fairly unspecific suppression of T-cells as well as other hematopoietic cells, which can also lead to severe side effects, particularly infections. We reviewed 1271 published studies (2011-2023) with regard to current LD strategies for approved anti-CD19 CAR-T products for large B cell lymphoma (LBCL). Fludarabine (Flu) and cyclophosphamide (Cy) (alone or in combination) were the most commonly used agents. A large number of different schemes and combinations have been reported. In the respective schemes, doses of Flu and Cy (range 75-120mg/m2 and 750-1.500mg/m2) and wash out times (range 2-5 days) differed substantially. Furthermore, combinations with other agents such as bendamustine (benda), busulfan or alemtuzumab (for allogeneic CAR-T) were described. This diversity creates a challenge but also an opportunity to investigate the impact of LD on cellular kinetics and clinical outcomes of CAR-T. Only 21 studies explicitly investigated in more detail the influence of LD on safety and efficacy. As Flu and Cy can potentially impact both the *in vivo* activity and toxicity of CAR-T, a more detailed analysis of LD outcomes will be needed before we are able to fully assess its impact on different T-cell subsets within the CAR-T product. The T2EVOLVE consortium propagates a strategic investigation of LD protocols for the development of optimized conditioning regimens.

## Background

Adoptive cell transfer (ACT) for the treatment of malignancies has become one of the most active and fruitful developments in therapeutic advances against cancer in the past 3 decades. Not so long ago, a series of studies lead by Dr. Rosenberg opened a new field by proposing to re-infuse *in vitro* expanded and IL-2-stimulated tumor-infiltrating lymphocytes to overcome immunotolerance in patients with metastatic melanoma and achieve tumour-control (1). Three decades later, translational research has further developed procedures, including direct activation of immune-effector cells against specific targets in an MHC-independent manner (2, 3). In this respect, T-cells modified to express chimeric antigen receptors (CAR-T) demonstrated the potential to achieve deep and durable remissions across a wide variety of hematological malignancies (4–27) and are now established as an important part of antineoplastic therapies in lymphoproliferative diseases (28–30). Expansion to new hematologic indications, solid tumors, autoimmune and infectious diseases is on the horizon (31). As of September 2023, there are six FDA and EMA approved products, improving the lives of children and adults with B-Cell Acute Lymphoblastic Leukemia (B-ALL), various forms of B-cell Non-Hodgkin Lymphoma (NHL) and Multiple Myeloma (MM). Many more therapies are currently going through different phases of clinical trials. Approved CD19-CAR-T therapies show high response rates in B-ALL and B-NHL, depending on the CAR-T product, line of treatment and specific indication, with unprecedented long-term progression-free and overall survival in patients with relapsed or refractory disease (11, 12, 14–16, 32) The same holds true for B-cell maturation antigen (BCMA) directed CAR-T for MM (24, 25), but unfortunately a considerable proportion of these (high-risk) patients still relapse after or are refractory to CAR-T therapy. Hence, there is still a need to improve efficacy of many steps in the CAR-T treatment sequence including T-cell quality, product characteristics, manufacturing, lymphodepleting regimens as well as post-treatment management.

Lymphodepletion (LD) has been identified as a critical factor playing a major role in the outcomes achieved with CAR-T treatment (33).

**Figure 1:**
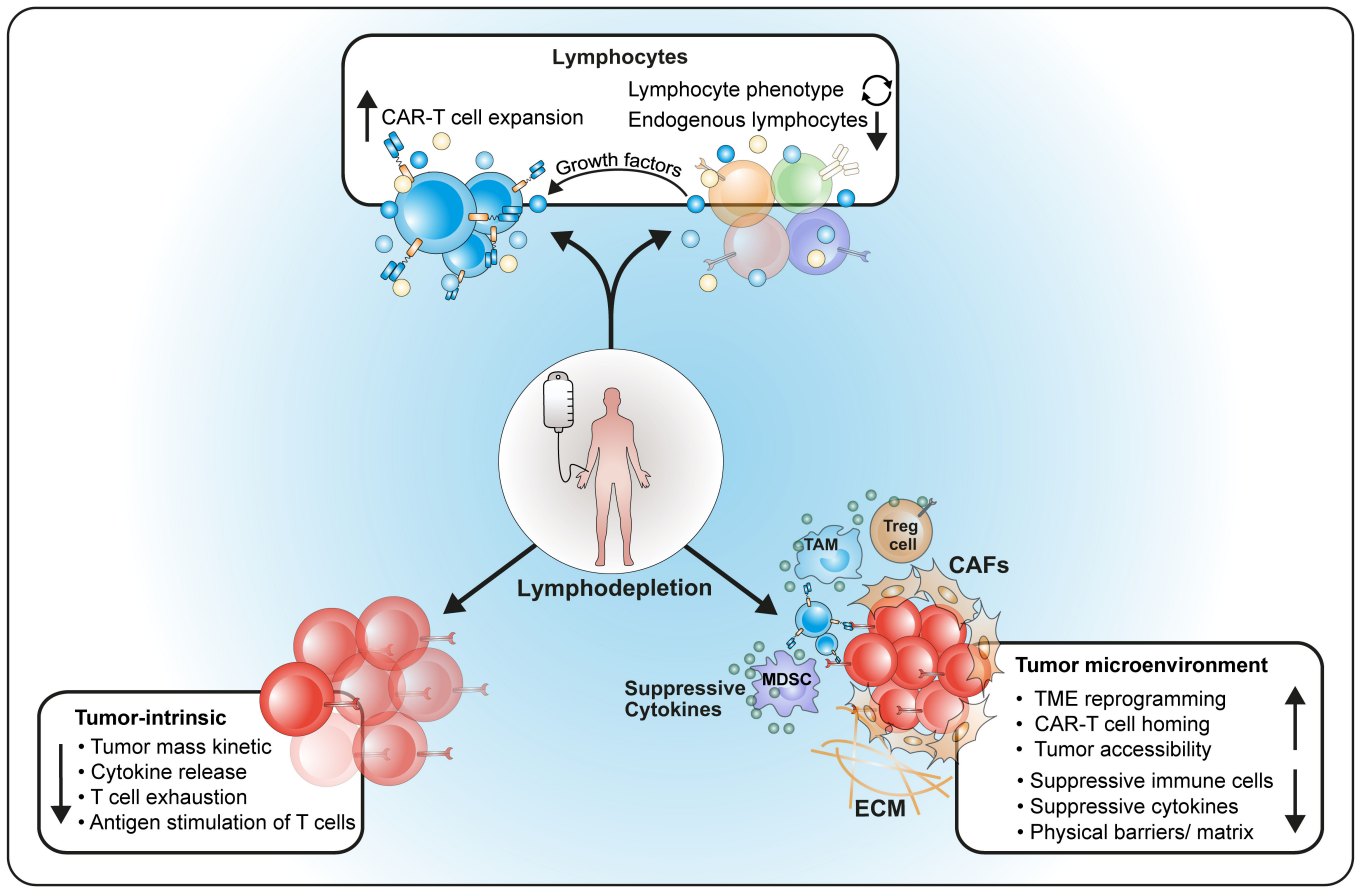
Major effects of lymphodepleting therapy on the host. LD influences (1) number and composition of host lymphocytes as well as cytokine production; (2) reduces the tumor and modulates its behavior; (3) reprograms the host microenvironment for better homing of CAR-T cells.

The main purposes of LD are (1) the reduction of endogenous lymphocytes to prepare a niche for engraftment of CAR-T infusions and to support their long-term activity; (2) the reduction of tumor cells to avoid rapid exhaustion of CAR-T; (3) preparation and reprogramming of the microenvironment and soluble factors to ensure optimal engraftment, homing and long-term survival of CAR-T (**Figure 1**).

This is the reason why chemotherapeutic agents with both cytoreductive activity against tumor cells as well as T-cells are commonly used in LD. The most common schedules for LD (Fludarabine (Flu), cyclophosphamide (Cy) and combinations thereof) are based on the paradigm of pre-conditioning in small trials with tumor-infiltrating lymphocytes (34, 35) as well as for allogeneic hematopoietic stem cell transplantation (36).

Differences in LD regimens and dosages depend on the targeting disease (e.g. ALL, NHL, MM or solid tumors) as well as on the T-cell source (autologous vs. allogeneic). However, while almost every patient receives LD, the actual evidence for their activity as well as the underlying experimental data are surprisingly scarce (37, 38).

Moreover, different LD regimens are usually linked to specific CAR-T products in use, making evaluations in regard to their influence on clinical outcome nearly impossible. This is a major obstacle encountered in head-to-head comparisons and meta-analysis.

Recent evidence from population-based pharmacokinetic studies suggests that efficacy and toxicity of LD differ even within the same regimen (37–39).

Therefore, studies investigating the activity and toxicity of LD in depth are still needed.

Focusing on malignant diseases, we here review the rationale and current evidence for various LD regimens and dosages, adjustments and novel approaches with the intention to facilitate selection of research topics for this important aspect of CAR-T therapy.

## Methods

This review is based on a systematic search of the literature, with the initial intention to perform a meta-analysis on LD.

The preliminary aim to identify publications on different doses of lymphodepletion was conducted on the 14.03.2023 via PubMed including the terms (“LYMPHODEPLETION” OR “CONDITIONING” OR “PREMEDICATION”), (“CART” OR “CAR-T” OR “CAR-T CELLS” OR “CHIMERIC ANTIGEN RECEPTOR T-CELLS”) and (“CD-19” OR “CD19”) delivering 21 studies of interest. While a more thorough search of those publications did not show the variety and comparability of lymphodepleting regimens, a broader analysis, including all 1271 published studies on approved anti-CD19 CART products for LBCL (axicabtagene ciloleucel, tisagenlecleucel and lisocabtagene maraleucel), only showed specific data on lymphodepleting regimens in 0.016% of those. A search of currently ongoing, planned or completed clinical trials was conducted on the 16.07.2023 using the search terms “CAR-T-Cells” and “Lymphodepletion” showed 171 results which are displayed in **Table 1**.

**Table 1:** Currently used lymphodepleting/conditioning regimens (26 variants).

Lymphodepletion Regimen	Dose (mg)	Dose total (mg)	Days	Timing	Comments / Reference	Variant
Fludarabine/ Cyclophosphamide	25/250/m^2^	75/750/m^2^	3	day -6 to -2	JULIET, PORTIA studies	1a
Fludarabine/ Cyclophosphamide	25/250/m^2^	75/750/m^2^	3	day -7 to -2	NCT05445011	1b
Fludarabine/ Cyclophosphamide	25/250/m^2^	75/750/m^2^	3	day -4 to -1	iPD1 CD19 eCAR T cells NCT03208556	1c
Fludarabine/ Cyclophosphamide	25/250/m^2^	75/750/m^2^	3	day -3 to -1	Anti-EGFRvIII CAR T Cells NCT02844062 NCT02937844	1d
Fludarabine/ Cyclophosphamide	25/300/m^2^	75/900/m^2^	3	day -5 to -3	CD19 CAR-T NCT05326243 CAR7-T Cells NCT04823091	2a
Fludarabine/ Cyclophosphamide	25/250/m^2^	75/750/m^2^	3		CI-135 CAR-T cells NCT05266950 JWCAR029 NCT05727683	2b
Fludarabine/ Cyclophosphamide	30/250/m^2^	90/750/m^2^	3	Day -5 to -3	NCT05326243 Dual CD33/CLL1 CAR T Cells NCT05248685 CD5 CAR T cells NCT05032599 CT125B NCT05487495	3
Fludarabine/ Cyclophosphamide	30/300/m^2^	90/900/m^2^	3	day-6 to -4	Varnimcabtagene autoleucel (ARI-0001), Cesnicabtagene autoleucel (ARI0002h), TranspoCART	4a
Fludarabine/ Cyclophosphamide	30/300/m^2^	90/900/m^2^	3	day -7 to -5	ciltacabtagene autoleucel CARTITUDE-1	4b
Fludarabine/ Cyclophosphamide	30/300/m^2^	90/900/m^2^	3	day -4 to -2	Idecabtagene vicleucel KarMMa huCART NCT03054298	4c
Fludarabine/ Cyclophosphamide	30/300/m^2^	90/900/m^2^	3	day -6 to -4	Lisocabtagene maraleucel TRANSCEND	4d
Fludarabine/ Cyclophosphamide	30/300/m^2^	90/300/m^2^	3,1	Day -5 to -3, Day -6	ISIKOK-19 NCT04206943	4e
Fludarabine/ Cyclophosphamide	30/300/m^2^	90/900/m^2^	3	CART 2-14d after LD	CAR.B7-H3 NCT04670068 ATLCAR.CD128 NCT03672318 NCT05634785 ATLCAR.CD30	4f
Fludarabine/ Cyclophosphamide	30/300/m^2^	90/900/m^2^	3		Anti-BCMA CAR-T Cell NCT04637269 CD19 and CD22 Dual-targeted CAR-T Cells NCT04303247 Anti-CD19 Allo-CAR-T Cells NCT04516551	4?
Fludarabine/ Cyclophosphamide	30/500/m^2^	90/1000/m^2^	3,2	day-5 to -3	ZUMA-7 OPBG PBCAR20A NCT04030195	5a
Fludarabine/ Cyclophosphamide	30/500/m^2^	90/1500/m^2^	3	day -5 to -3	Axicabtagene Ciloleucel Routine, ZUMA-1, CARTITUDE, KaRRMa, JCAR017/TRANSCEND, MB-CART20.1, MB-CART2019.1 (lymphoma)	5b
Fludarabine/ Cyclophosphamide	30/500/m^2^	90/1500/m^2^	3	day -7 to -5	Bexucabtagene autoleucel ZUMA - 2	5c
Fludarabine/ Cyclophosphamide	30/500/m^2^	90/1500/m^2^	3	CART 2-14d after LD	NCT02690545 NCT02917083	5d
Fludarabine/ Cyclophosphamide	30/500/m^2^	90/1500/m^2^	3	day -4 to -2	NCT02443831 CARPALL UF-KURE19 NCT05400109 CARTinNS NCT04561557 CT125A cells NCT04767308	5e
Fludarabine/ Cyclophosphamide	30/500/m^2^	120/1000/m^2^	4,2	Completed at day -2	NCT05010564 TRICAR-ALL ELIANA NCT03321123	5f
Fludarabine/ Cyclophosphamide	30/500/m^2^	150/1500/m^2^	5, 3	day -7 to -3, day -4 to -2	CD19/22 CAR T-cells NCT02443831	5g
Fludarabine/ Cyclophosphamide	30/500/m^2^	90/1500/m^2^	3	day -5 to -3	CD22 CAR NCT04088890	5h
Fludarabine/ Cyclophosphamide	30/500/m^2^	120/1000/m^2^	4,2	CART 1-2d after LD	Anti-GPC3 CAR T NCT02876978	5i
Fludarabine/ Cyclophosphamide	30/750/m^2^	90/750/m^2^	3,1	Day -5 to -3, Day -5	fhB7H3.CAR-Ts NCT05211557 B7H3 CAR-T Cells fhB7H3.CAR-Ts NCT05323201	6a
Fludarabine/ Cyclophosphamide	30/750/m^2^	90/750/m^2^	3	day -6 to -4	NCT05950802 ODIN	6b
Fludarabine/ Cyclophosphamide	25/900/m^2^	75/900/m^2^	3,1	Day -4 to -2, Day -2	NCT04088864 Bexucabtagene autoleucel ZUMA - 2	7
Fludarabine/ Cyclophosphamide	25/m^2^/30mg/kg	75/m^2^/90 mg/kg	3	Complete at day -2	NCT00902044	8
Fludarabine/ Cyclophosphamide	20-30/300-500/m^2^	80-120/600-1000/m^2^	4,2		CD19 CAR T-Cell(CAT19T2) NCT05613348	9
Fludarabine/ Cyclophosphamide	25/60mg/kg	75/180 mg/kg	3	day -7 to -5	F.Hutchinson	10
Fludarabine/ Bendamustine/ (Cyclophosphamide if hypersensitive to Bendamustine)	30/70/(300)/m^2^	90/210/(900)/m^2^	3	CART 2-14d after LD	ATLCAR.CD30 NCT04083495	11
Fludarabine/ Bendamustine	30/70/m^2^	90/210/m^2^	3	Day -5 to -3	CHARIOT-Trial NCT04268706	12
Fludarabine/ Busilvex	40 mg/m^2^ /3.2 mg /kg	160 mg/m^2^ /12,8 mg /kg	4	Day 1-4	INSERM	13
Fludarabine/ Cyclophosphamide/ Alemtuzumab	90 mg/m^2^ ; 1500mg/m^2^ ; 1mg/kg or 40mg				Servier - Adult trial	14
Fludarabine/ Cyclophosphamide/ Alemtuzumab	150mg/m^2^ ; 120mg/m^2^ ; 1mg/kg (capped at 40mg)				Servier - Pediatric trial	15
Fludarabine/ Cyclophosphamide/ TLI	30/500/m^2^ 2Gy in 2 fractions	90/1000/m^2^ 2Gy in 2 fractions	3,2	day -6 to -4 TLI day -3 to -2	NCT05950802 ODIN	16a
Fludarabine/ Cyclophosphamide/ TLI	30/750/m^2^ 2Gy in 2 fractions	90/1500/m^2^ 2Gy in 2 fractions	3,2	day -6 to -4 TLI day -3 to -2	NCT05950802 ODIN	16b
Fludarabine/ Cyclophosphamide/ ALLO-647					NCT04416984 ALPHA2	17
Fludarabine/ Cyclophosphamide/ Etoposide					NCT05776407	18
Fludarabine/ Cyclophosphamide/ VP-16					NCT05679687 NCT05640713 NCT05691153 NCT05576181 ThisCART19A	19
Fludarabine/ Cyclophosphamide/ Etoposide/ Cytarabine/ Dexamethasone	24/150/90/180/6/m^2^	120/750/450/900/30/m^2^	5		NCT04499573	20
Fludarabine/ Cyclophosphamide/ Nirogacestat					NCT04171843	21
Cyclophosphamide	1800/m^2^	3600/m^2^	2		anti-GD2 CAR T cells NCT02107963	22
Cyclophosphamide	1500/m^2^	1500/m^2^	1	Day -3	CART-meso cells NCT03638193	23
Cyclophosphamide	300/m^2^	900/m^2^	3	Day -5 to -3	αPD1-MSLN-CAR T Cells NCT04489862	24
Bendamustine	90/m^2^	180/m^2^	2	day-6 to -2	Tisagenlecleucel Routine, JULIET, PORTIA studies	25
Alemtuzumab					NCT04150497 BALLI-01	26

It became clear that due to the lack of conclusive data and the close link of individual LD regimens to specific CAR-T products, a meaningful meta-analysis of response and toxicity is currently not feasible.

Thus, instead of a meta-analysis of LD regimens, members of the T2EVOLVE work package 6 presents here a comprehensive literature review.

## Currently used substances and lymphodepleting regimens

**Table 1** shows the great variety of currently applied LD/conditioning regimens across clinical trials. These treatments differ in substances and their combinations, dosing and timing (even within LD regimens with identical doses). The regimens also differ between autologous and allogeneic CAR-T therapies. Depending on the indication (lymphomas, leukemias or solid tumors), various approaches have been proposed. The following substances or combinations thereof are the main stay of LD:

### Fludarabine

Fludarabine (Flu) is a purine analogue, which has been a vital treatment option for Chronic Lymphocytic Leukemia (CLL) and indolent NHL for many years (40), is mainly used in combination with other lymphodepleting agents. Activity and toxicities are well studied in CLL and conditioning for allogeneic stem cell transplantation (41). Fludarabine seems to be particularly toxic for hemopoietic precursors – a fact that may account for some early toxicity after infusion (42, 43). The metabolism of the drug is dependent on glucuronidation by the UGT2B17-pathway, resulting in dependency on individual, sex- and ethnicity-related factors (44).

### Cyclophosphamide

Cyclophosphamide (Cy) is an alkylating agent with efficacy in a wide range of lymphomas, Hodgkin´s lymphoma, aggressive as well as indolent NHLs (45–47). High-dosage Cy is used as a single lymphodepleting agent followed by CAR-T transfusion or in autologous stem cell transplantation. Dosages in the CAR-T setting up to 1800 mg/m^2^ for two days have been studied for efficacy (33).

### Fludarabine and cyclophosphamide

Flu and Cy are used in combination with immunotherapy for CLL (48) and are currently the most commonly used lymphodepleting agents used for CAR-T cell therapy. Most LD regimens are based on the doses used for CLL therapy (49). Commercially available CAR –T products rely mostly on Flu + Cy for conditioning, except for tisa-cel (11) (**Table 1**). However, variations in dosages of Flu/Cy (25/250 mg/m^2^ up to 30/750 mg/m^2^) application duration (3 days up to 5 days of Flu and 1 up to 3 days of Cy respectively) and time until transfusion (1 day up to 14 days prior to transfusion) of CAR-T are common.

### Bendamustine

Another alkylating agent is Bendamustine (Benda), used as a first- and second-line treatment option for CLL and other B- and T-cell lymphomas, either as single agent or in combination with targeted therapies (50–52).

While being included as an alternative to Flu/Cy in the JULIET trial (11) and showing fewer toxicities than Flu/Cy with similar outcomes (53), it is not included as an option for other products and only occasionally used in clinical trials regarding novel CAR-T. B seems to be a good option for patients with renal insufficiency (53).When used in clinical trials the dose ranges from 70 mg/m^2^ to 90 mg/m^2^ per day, for two consecutive days, as a single agent.

### Radiation

Radiation therapy (RT) is nowadays used in the treatment of localized lymphomas (54–56), as well as for transplant conditioning. Radiation of tumor masses shows benefits in animal studies as a pre-conditioning before CAR-T administration enabling them to mitigate antigen escape (57). In preclinical mouse models, total body irradiation has been used for a long time to induce lymphodepletion. In addition, low dose local radiation in combination with CAR-T is currently a promising field especially in the context of solid tumor therapies.

### Targeted lymphodepletion

#### Alemtuzumab

As an alternative, targeting antigens on T-cells (and malignant cells) specifically has been explored. Alemtuzumab, alone or in combination with Flu/Cy is being used as conditioning agent for allogeneic CAR-T, particularly in lymphoproliferative diseases, where it seems to improve the clinical response rate over Flu/Cy (58). Higher toxicities should, however, be expected (58, 59).

Moreover, alemtuzumab due to its long half-life and pan-T activity can only be applied when CD52 has been knocked out in CAR-T.

Most of the regimens target both B as well as T-cells, making them particularly attractive to combine LD as well as tumor reduction in lymphomas. On the other hand, metabolism, route of elimination and half-life are different and may even vary between ethnicities. This may account for varying toxicities and efficacy (60). Serum concentrations as well as population genetic differences have been linked to these effects (39, 61, 62).

Differences in the total dose or effective serum levels of Flu/Cy may even account for outcome. It is interesting to note that tisa-cel which uses a lower dose of Flu/Cy has shown lower long-term progression-free survival and is the only agent failing in second-line trials (11, 13, 23, 63).

Variations in the interval between LD and CAR-T delivery may also be responsible for differences in toxicities and could even affect the growth and cellular kinetics of CAR-T, since very close timing to infusion may be associated with considerable serum concentrations of fludarabine after day+1 in special populations (39, 61, 62). Moreover, T cell subsets show different sensitivity to Flu, especially CD8^+^ effector T cells seem to be more sensitive (64). Therefore, exploring options targeting T cells or subsets thereof seems warranted.

#### Oxaliplatin/cyclophosphamide

This combination has been reported to enhance recruitment of CAR-T to solid tumors and to increase efficacy when combined with checkpoint blockade (65, 66).

#### Clofarabine

Clofarabine is well known for its activity as an anti-leukemic agent. It has successfully been used for LD together with cyclophosphamide before tisa-cel CD19+ CAR-T cell infusion leading to clinical remission (67, 68) Another potential indication is its use as conditioning for CAR-T reinfusion as in the case of ARI-0001 (22). Flu/Cy plus rituximab has been used for conditioning before a second CAR-T infusion (69).

### Emerging strategies to avoid the need for LD

Several studies have demonstrated the potential of alternative strategies to enhance the *in vivo* activity of immune effectors without the need for lymphodepletion in CAR-T cell therapy. These strategies aim to overcome the limitations of lymphodepletion.

Such approaches seek to enhance CAR-T cell persistence and expansion by incorporating additional gene engineering besides the transfer of the CAR gene construct. This can be achieved by engineering CAR-T cells to also express certain cytokines that can help promote their survival and proliferation. These are considered “4th generation” CAR-T cells, or TRUCKs (“T cells redirected for antigen-unrestricted cytokine-initiated killing”) (70), an approach with a particular interest in solid tumors. Currently there are several TRUCK designs being tested in preclinical and early phase trials testing the incorporation of a wide variety of single or combinatory cytokines with autocrine and paracrine functions including IL-7, IL-12, IL-15, IL-18, and IL-23, among others.

Other complementary gene engineering candidates that could potentially allow for enhanced CAR-T cell persistence and expansion can also include the intervention of immune-checkpoints like PD-1 and CTLA-4. This could be achieved by either the incorporation of the capacity to secrete PD-1 and CTLA-4 blocking antibodies directed upon antigen-driven CAR signaling, or by directly disrupting PD-1 gene (71), an approach that potentially could prevent T-cell exhaustion and/or suppression.

Nevertheless, while these approaches may seem promising, they are still in very early stages of development, and all currently commercially available CAR-T cell products strongly rely on preconditioning, highlighting the need to identify optimal LD regimens to improve the safety and efficacy of adoptive cell therapy with engineered T-cells.

## Evidence and rationale for dosing in humans

Lymphodepleting chemotherapy has a 3-decade long history of co-development with adoptive cell transfer. Right from the start, LD has proven to be of upmost importance as it drives a synergistic effect that outperforms the expected effect of what both, chemotherapeutic agents and immune-effector cells would achieve by themselves. In fact, initial reports in murine models showed how the prior administration of cyclophosphamide facilitated the elimination of an established tumor by eliminating tumour-induced suppressor T-cells (72). By doing this, the administration of cyclophosphamide unveiled the importance of reducing immunosuppression to boost T-cell mediated immunity. Thereby it was shown that cyclophosphamide administration could also unleash an antitumoral effect that was not directly related to LD itself.

While non-myeloablative conditioning prior to ACT significantly improves the efficacy of tumor-reactive T cells in pre-clinical models, complete ablation with higher conditioning doses can intensify these effects. In a non-myeloablated host the regulatory compartment is significantly reduced with increased cytokine availability of homeostatic cytokines like IL-7 and IL-15, which positively impacts the anti-tumor efficacy of adoptively transferred T cells. An intensified lymphodepletion up to the point at which HSC reconstitution is required, further promotes to the beneficial environment. Studies in the pmel-1 model for the treatment of established B16 melanoma showed that intensified myeloablative pre-conditioning up to 9 Gy in combination with HSC improves the efficacy by further reduction of homeostatic cytokine consuming host cells (73, 74). Nevertheless, the administration of higher conditioning intensities has to be balanced out with emerging toxicities (34).

The significance of conditioning intensity was further examined in the early stages of TIL therapy among patients with metastatic melanoma (75). Ninety-three participants with metastatic melanoma underwent TIL therapy following three distinct conditioning regimens in three non-randomized sequential studies. These regimens included a non-myeloablative chemotherapy-based approach with Cy/Flu (n=43) and two myeloablative regimens incorporating Cy/Flu along with either 2 or 12 Gy of TBI (both n=25). Based on previous studies in murine models indicating enhanced TIL efficacy with increased conditioning intensity reaching a myeloablative level, followed by autologous hematopoietic stem-cell rescue (34, 74), the clinical study (75) reported overall response rates of 48.8%, 52%, and 72% for non-myeloablative, 2-Gy myeloablative, and 12-Gy myeloablative conditioning, respectively. Additionally, complete response rates were 9.3%, 8%, and 16%, respectively. Despite the limitations of non-randomized sequential studies, a discernible positive impact on efficacy appears notable in patients conditioned with high-TBI myeloablative protocols.

### Influence on the microenvironment

It was not long before the foundations of LD were laid, as it was discovered that most of the synergistic effects were not only mediated by the direct antitumoral effect itself, but rather on how LD modulates the environment in which the immune system acts (76, 77). Soon, the identification of several mechanisms on how LD was able to modulate the activity of transferred immune-effector cells was initiated, including its modulation of homeostatic cytokines such as IL-7 and IL-15 by abrogation of cellular cytokine ‘sinks’, or other immune cells that compete with transferred immune-effector cells for the consumption of stimulatory cytokines. Also, the impairment of regulatory T-cells (Treg) that suppress tumor-reactive T-cells contributes to polarization of the environment. Finally, the induction of tumor apoptosis and necrosis can potentially lead to an increased cellular immunity, driven by an increased tumor-antigen presentation. These findings were obtained when connecting increased tumor-regression with the absence of host lymphocytes. In fact, a link between LD and augmented immune function is suggested to be mediated by `homeostatic proliferation` (78–85), a process that drives increased T-cell proliferation after small numbers of them are transferred into a lymphopenic host (see also chapter pre-clinical experimental evidence). Evidence for the impact of competition for homeostatic cytokines between transferred immune-effector cells and host cells (or cellular cytokine ‘sinks’) has been described (78, 80, 85, 86), as the proliferation of transferred adoptive T-cells in lymphopenic hosts can be reduced in a dose-dependent manner by increasing the total number of either adoptive antigen-specific T-cells or by co-transferring an ‘irrelevant’ population of T-cells. Furthermore, the eradication of ‘cellular sinks’ by LD and the resultant increase in the availability of homeostatic cytokines has been described as a central mechanism driving the activation of antigen-specific CD8^+^ T cells (77). Finally, naturally occurring Treg can potently induce immunotolerance to self and foreign antigens, as the physiological function of Treg is thought to be the avoidance of autoimmunity by ensuring tolerance to self-antigens. Therefore, a close link between autoimmunity and tumor immunity suggests that Treg may have a crucial role in shaping a tolerogenic TME (87).

Intestinal microbiota are also involved in modulating the activity of cellular therapies including CAR-T cells. In addition to the LD regimen itself, antibiotic treatment as prophylactic intervention does cause dysbiotic states. Experimental approaches have shown that the microbial composition and commensal-derived metabolites crucially impact on host immunity, thereby shaping either tolerogenic or activating environments (88, 89). Further, recent studies have shown that the microbiome is capable of influencing the functionality of CD19-specific CAR-T cells in patients. Those correlated the use of antibiotics before CAR-T cell administration with decreased commensal diversity and worse survival highlighting a beneficial role of a rich microbiome in cellular therapy (90, 91). In that regard, it was demonstrated that commensal metabolites such as short-chain fatty acids might improve the function of adoptively transferred T-cells for cancer treatment (92). Interestingly, LD can also contribute to anticancer reactivity by causing bacterial translocation to other tissues than the intestine itself such as the mesenteric lymph nodes. There, activation of antigen-presenting cells by TLR ligands can enhance the stimulation of adoptively transferred T-cells with antitumoral activity (93). Translocation of commensals to the tumor site has been described as well. By local secretion of L-lactate, *Lactobacillus iners* is able to confer chemoradiation resistance in mouse and patients (94). In contrast, the related strain *Lactobacillus reuteri* releases a tryptophan metabolite intratumorally, thereby improving checkpoint inhibition (95). Hence, both the translocation of the microbiome to other sites as well as the modulatory capacity of bacterial metabolites should be considered as potential factors altering immunotherapy response which are in turn changed by LD and antibiotic regimens.

### Reduction and modulation of lymphocytes

Further clinical evidence of the relevance of LD has been previously described in early trials of tumor-infiltrating lymphocytes (TIL) on patients with metastatic melanoma, where a LD regimen consisting of 2 days of cyclophosphamide 60 mg/kg followed by 5 days of 25mg/m2 fludarabine could effectively reduce leukocyte counts to fewer than 20 cells/mm3, allowing for TIL administration in a lymphodepleted status (35). Furthermore, this transferred adoptive immune-effector cell expansion could be boosted by the administration of the cytokine IL-2 (96). Similar outcomes were observed in another early TIL trial for patients with metastatic melanoma, where 10 patients received TIL treatment in two occasions, the first one without LD, and the second one following 5 days of fludarabine 25mg/m2, allowing for intra-patient comparison (97). Investigators observed that prior administration of fludarabine led to a 2.9-fold increase in the *in vivo* persistence, as well as also led to an increase in plasma levels of the homeostatic cytokines IL-7 and IL-15.

The importance of LD was later evaluated with the first clinical reports of administration of CAR-T, where a small group of patients received treatment with a first generation (CD3z) CD20-specific autologous CAR-T in the absence of LD. In the trial reported by Till and colleagues (98) the first 3 patients had a very limited expansion and persistence of the transduced cells (1-3 weeks) that was later increased up to 9 weeks after the addition of IL-2 administration. Due to the modest therapeutic effects observed in this trial, the construct was modified through the addition of two costimulatory domains (CD28-4-1BB-CD3z) and LD with 2 days of cyclophosphamide 1000 mg/m2 was followed by IL-2 administration (99). Using these modifications, the persistence of the transduced cells was detectable for up to 12 months, and 2 out of 4 patients achieved a complete response (CR). Consequently, at the nadir of LD, the absolute Treg levels, absolute CD3^+^ counts, and B cell counts were reduced by up to 96%, 93%, and 85%, respectively. However, despite the rapid lymphocyte expansion observed during IL-2 therapy, a possible counterproductive effect was detected as increased Treg levels were also seen after IL-2 injections. Therefore, IL-2 administrations were abandoned. Similar outcomes were observed in another early CAR-T report (100), this time with a more sophisticated construct composed of an anti-CD19 (FMC63), CD28-CD3z autologous product administered to a patient with lymphoma. The patient received an LD regimen consisting of 2 days of cyclophosphamide 60 mg/kg followed by 5 days of fludarabine 25 mg/m2. B cell aplasia has been observed for more than 3 years and the patient achieved a dramatic remission that lasted 32 weeks.

LD with Flu/Cy leads to changes in cytokine profiles and lymphocyte composition of the host, particularly when applied with growth factors such as GM-CSF (101). CD4+ T regulatory cells, CD8+ T suppressor cells, and T memory cells (CD8+ T central memory cells; T effector memory RA+ cells) are increased. Long-term changes of the T-cell and cytokine profiles are also observed with bendamustine (102).These long-term immunomodulatory effects of LD probably facilitate the development of opportunistic infections (103).

### Reduction of rejection

The importance of the achievement of lymphodepletion was suggested in another early CAR-T trial (104) that observed how anti-transgene rejection responses contributed to limited persistence and efficacy of a first generation, dual (CD20/CD19 specific) CAR-T in a small group of 4 patients with Relapsed/Refractory (R/R) DLBCL and Follicular Lymphoma (FL). CAR-T were infused either following autologous Hematopoietic Stem Cell Transplantation (HSCT) or before fludarabine administration. Detection of transduced cells was reduced to a week or less, and anti-transgene immune rejection responses were detected in half of the patients. No patients were infused while lymphocyte counts were below normal range. This unveiled the possible role of immune rejection of the transgene as a major driver in the engraftment of the transferred cells, which in turn raised the interest in the immunosuppressive (IS) power of LD: an intensified LD might also lead to increased immunosuppression, consequently improving engraftment by avoiding anti-CAR-T-specific response.

### Consolidation of efficacy

After these initial observations, several groups started reporting encouraging response rates and engraftment of CAR-T in patients with R/R B-ALL. A trial treating 16 adult ALL patients with Cy 1.5 to 3.0 g/m2 as LD followed by an anti-CD19 CD28-CD3z was able to achieve an unprecedented complete response rate (CRR) of 78% (105). Similar outcomes were observed in the pediatric setting in another trial (106) in which 30 patients were treated with a wide variety of LD regimens (Flu/Cy, Cyclophosphamide, Vincristine, and Prednisone [C/VP] and Cyclophosphamide, Vincristine, Doxorubicin, Dexamethasone [CVAD]) followed by CTL019 (later known as tisa-cel). In this trial, the CRR was 90%, with a 6-month rate of EFS and CTL019 persistence of 67% and 68%, respectively. Interestingly, most of the patients received LD, with at least half of them receiving Flu/Cy. Finally, a third trial (107) for R/R ALL treated 14 additional patients with LD consisting of 3 days Flu 25 mg/m2 and 1 day of Cy 900 mg/m2 followed by an anti-CD19 CD28-based CAR-T, achieving an 85% Minimal Residual Disease (MRD)-negative CRR.

### Clinical evidence for the impact of fludarabine

Following the experience on the use of fludarabine in the context of allogeneic stem cell transplantation, new studies were designed to formally evaluate its impact on the LD regimen of patients with R/R ALL (108). On this trial, patients received a defined CD4^+^:CD8^+^ CAR-T composition with prior LD, either with Cy 2–4 g/m2 alone, Cy 2–3 g/m2 plus etoposide 100 mg/m2/d for 3 days, or Cy 60 mg/kg plus Flu 25 mg/m2/d for 3 or 5 days. Interestingly, patients receiving a fludarabine-based LD had improved CAR-T expansion, persistence and consistently with the increased CAR-T expansion, an improved disease-free survival.

Comparable outcomes were also observed in the pediatric setting in a study with 45 patients with R/R ALL treated with a defined CD4^+^:CD8^+^ CAR-T composition (4). Patients received LD either with cyclophosphamide alone or in combination with fludarabine. Patients that received Flu/Cy-based LD had a higher expansion peak, with a longer duration of B-cell aplasia in comparison to the ones that received Cy-based LD without Flu (2.1 vs 6.4 months). Finally, MRD-negative CRR was 100% and 90% for patients receiving LD with and without fludarabine.

Similar outcomes were obtained for patients with B cell lymphomas (109). In this study, patients who received Flu/Cy LD had a markedly increased CAR-T expansion and persistence, as well as higher response rates in comparison to the ones that received only cyclophosphamide-based LD (CRR of 50% vs 8%). Flu/Cy-based LD also led to higher serum concentrations of IL-7 and IL-15 on the day of CAR-T infusion. Furthermore, patients treated with cyclophosphamide based LD (without Flu) had an increased anti-CAR-T immune response, as second CAR-T administrations led to a lack of engraftment or measurable efficacy, as well as a detection of cytotoxic T cell responses specific for the murine single-chain fragment variable encoded by the transgene in 5/5 patients analyzed. This contrasts with the fact that 3/4 patients that received a second CAR-T infusion after previous exposure to Flu/Cy LD at their first CAR-T treatment were able to achieve a second engraftment which translated into tumor regression. These observations point towards to the importance of fludarabine to reduce the immune response to the transgene, allowing for longer persistence, efficacy, and eventually, for repetitive dosing of CAR-T cells.

Comparable outcomes were observed in a new study that focused on factors associated with durable event-free survival (EFS) in B-ALL patients after treatment with anti-CD19 CAR-T therapy (7). A total of 53 patients treated with a 4-1BB-based second generation product after being lymphodepleted with a variety of LD schemes. In this study, 85% of patients achieved a MRD-negative CR, and the multivariate modeling showed that the performance of LD regimens that incorporated fludarabine was associated with better EFS with a hazard ratio of 0.34. Interestingly, all CD19 negative relapses were observed in patients that received a fludarabine-based LD, and all relapses observed in patients whose LD did not contain fludarabine were CD19 positive. All this, together with the fact that a higher CAR-T AUC was seen in the Ful-based LD patients, is consistent with the fact that the magnitude of CAR-T expansion and persistence (and the LD performed) might influence the relapse phenotype.

Similarly, another study performed in 34 patients with R/R Hodgkin lymphoma analyzed the safety and efficacy of an autologous CAR-T targeting CD30 (27). In this trial, 3 different LD regimens were administered: Cy 500 mg/m2/day plus Flu 30 mg/m2/day for 3 days, bendamustine 90mg/m2/day for 2 days alone, and Benda 70 mg/m2/day and Flu 30 mg/m2/day for 3 days. Surprisingly, CRR varied between LD regimens to a point of achieving a 0% CRR in patients undergoing LD with Benda alone, which increased to 47% and 73% in patients lymphodepleted with Flu/Cy and Benda/Flu, respectively. These findings suggest that fludarabine seems to remain a fundamental drug in CAR-T LD therapy, which does not appear to be limited to products targeting B-cell malignancies.

### The issue of intensity – influence on CAR-T cell kinetics

Focusing on dose intensity, another study further analyzed the impact of increasing the intensity of Flu/Cy-based LD in patients with B-cell lymphomas (33). In this study, patients with R/R aggressive B-cell lymphomas were treated with an anti-CD19 CAR-T product following 5 different LD categorized into ‘high-intensity’ vs ‘low-intensity’ LD based on the dose of Cy administered. ‘High-intensity’ LD consisted on Cy 60 mg/kg with either 3 or 5 days of Flu 25 mg/m2/day. On the other hand, ‘Low-intensity’ LD consisted on Cy 30 mg/kg or with 3 days Flu 25 mg/m2/day, 3 days of Cy 300 mg/m2/day with 3 days Flu 30 mg/m2/day, or 3 days of Cy 500 mg/m2/day with 3 days Flu 30 mg/m2/day (**Table 1**).

**Figure 2:**
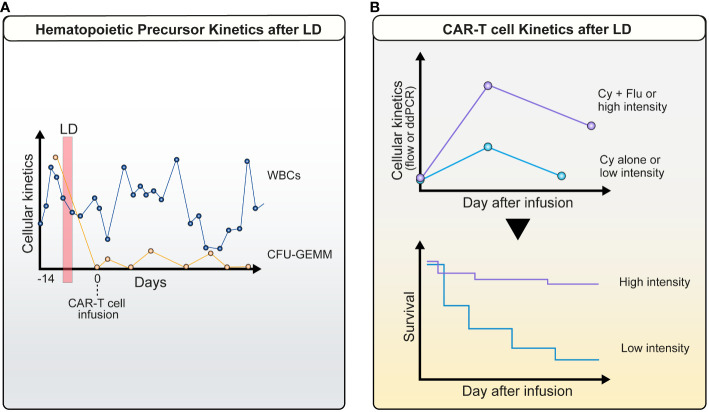
Cellular kinetics after lymphodepletion: **(A)** Profound decrease in host PB hematopoietic precursor cells. A marked drop in common myeloid progenitors CFU-GEMM (orange line) is observed between LD (red vertical bar) and CAR-T infusion in parallel with PB leukocyte counts (blue line) (patient example); **(B)** Kinetics of CAR-T cells is dependent on the intensity of LD. This is accompanied by an improved survival.

In this study, the best ORR was 51%, with 40% of patients achieving a CR and a 46% probability of estimated 2-year Progression Free Survival (PFS). However, patients that received ‘high-intensity’ LD achieved a more favorable cytokine profile, as defined by the levels of monocyte chemoattractant protein-1 (MCP-1) prior to infusion and peak interleukin-7 (IL-7). Those concentrations were above the median in patients that had a “high-intensity LD”, which in turn translated into higher CAR-T peak expansion, higher Cytokine Release Syndrome (CRS) intensity and severity, and a superior PFS (**Figure 2B**).

Nevertheless, an increase in intensity of LD might not always be associated with increased efficacy, as found in another study performed in R/R ALL (110)). 50 CAYA (children, adolescents, and young adult) patients were treated with a CD28-based anti-CD19 autologous CAR-T with the option of choosing the intensity of the LD regimen based on the disease burden observed prior infusion. This approach was designed to test the hypothesis that alternative LD regimens could reduce disease burden better prior to CAR-T infusion, with the objective of reducing CRS severity and potentially improve efficacy. ‘High-burden’ disease was defined by >25% bone marrow blasts, circulating peripheral blasts, or lymphomatous disease. Patients with ‘high-burden’ tended to receive an increased intensity LD either with (1) high-dose Flu (30 mg/m2 once a day x4 days) plus Cy (1,200 mg/m2 once a day x2 days), (2) FLAG, Flu (25 mg/m2 x 5 days) and cytarabine (2,000 mg/m2/day x5 days); and (3) ifosfamide (1,800 mg/m2 once a day x5 days) plus etoposide (100mg/m2 once a day x5 days), whereas patients with ‘low-burden’ disease received ‘standard-dose’ LD consisting of Flu (25 mg/m2 x 3 days) and Cy (900 mg/m2 x1 day). As for efficacy, MRD-negative CR was achieved in 56% of patients. CRR tended, however, to be significantly lower in patients that received increased intensity LD, with only 25% of patients achieving CR, which compares unfavorable with the 69% CRR observed in patients that received Flu/Cy-based LD. These outcomes suggest that increasing the intensity of LD might not be enough to overcome the bad prognosis associated with high-tumor burden.

All studies mentioned above indicate that the search for an optimal dose for LD for CAR-T rather reflected a ‘trial and error ‘strategy based on many drugs used in allogeneic stem cell transplantation and was not based on actual pharmacokinetic studies. Over the last decade, however, multiple studies showed a major impact of the individual pharmacokinetics for Anti-Thymocyte Globulin ATG (111), busulfan (112) but also fludarabine (61, 113) on the immune reconstitution of transplanted patient, leading to a proposal for optimal fludarabine dosages to be used (15-25 mg*h/l) to allow a well-balanced immune reconstitution with high efficacy and low toxicity. Therefore, prospective clinical trials are underway to test the impact of individual fludarabine dosing on immune reconstitution after allogenic stem cell transplantation (EudraCT Number: 2018-000356-18). Within this context, hypothesis-generating retrospective studies have been performed to assess the impact of fludarabine on CAR-T engraftment and clinical outcome, suggesting that intra-individual pharmacokinetics are an important factor to consider, even if patients receive at the first sight a similar dose. In the first study (114), CAYAs treated with commercial tisa-cel with prior on-label ‘conventional’ Flu/Cy LD had their cumulative total fludarabine exposure (area under the concentration-time curve [AUC]) measured. Blood samples were taken at time 0, + 1, +3, +7 and +11 hours after infusion of fludarabine on days 1 to 4 of LD. Concentrations of the circulating fludarabine metabolite were measured by a validated mass spectrometry method. Given the different needs of LD for allogeneic stem cell transplantation and CAR-T infusion, focus in this study was mainly on underexposure, as creating space for infused immune cells is the main goal. Patients were retrospectively categorized into ‘underexposure’ if the area under the AUC of fludarabine was lower than 14 mg*h/L. In the underexposed group, the median leukemia-free survival (LFS) was 1.8 months, which was lower compared with the 12.9 months observed in the group with an AUC of F ≥14 mg*h/L. Furthermore, the duration of B-cell aplasia within 6 months was shorter in the underexposed group (77.3% vs 37.3%). In addition, the cumulative incidence of CD19-positive relapse within 1 year following infusion was 100% in the underexposed group, which was significantly higher compared with 27.4% in the group with an AUC ≥14 mg*h/L. Finally, higher fludarabine exposure was associated with increased CAR-T expansion within the first 28 days after CAR T-cell infusion, with a mean peak CAR-T expansion in Peripheral Blood (PB) of 102 CAR-T cells/mL in the underexposed group vs the 295 CAR-T cells/mL in the group with an AUC ≥14 mg*h/L.

Comparable outcomes were observed by a second study that estimated the fludarabine exposure also as an AUC using a validated pharmacokinetic model in CAYAs treated with commercial tisa-cel with on-label, conventional’ Flu/Cy LD (62)). Fludarabine exposure was related to overall survival (OS), cumulative incidence of relapse (CIR), and a composite end point that included loss of B-cell aplasia and/or relapse. In this study, optimal fludarabine exposure was also similarly determined as an AUC ≥13.8 mg × h/L. In multivariable analyses, patients with AUC <13.8 mg × h/L had a 2.5-fold higher CIR and two-fold higher risk of relapse or loss of B-cell aplasia compared with those with optimal fludarabine exposure.

Similar outcomes were seen in patients with aggressive B-cell lymphomas treated with commercially available axicabtagene ciloleucel (115). In this study, a retrospective analysis of the impact of the fludarabine exposure on the safety and efficacy of axi-cel was measured in 199 adult patients. By estimating the fludarabine AUC using a validated pharmacokinetic model, three AUC categories were identified based on the results of P-splines curves: low (<18 mg*h/L), optimal (18-20 mg*h/L), and high (>20 mg*h/L). As previously described with tisa-cel in B-ALL, optimal fludarabine AUC was significantly associated with the most favorable PFS and OS. The 12-month PFS rate was 66% for the optimal AUC, which compared favorable to the 39% and 46% observed in the low and high groups, respectively. Also, the 12-month OS rate was 77% for the optimal AUC, which compared favorable to the 59% and 66% observed in the low and high groups, respectively. Given the different needs of LD for allogeneic stem cell transplantation and CAR-T infusion, focus in this study was mainly on underexposure, as creating space for infused immune cells is the main goal.

## Animals/preclinical – experimental evidence

Murine model systems remain a powerful tool for the functional evaluation of adoptive cell therapy with immune-effector cells.

Xenograft models allow the transplantation of human tumor cells and primary human T cells to study the anti-tumor efficacy. To enable the successful engraftment of human cells, highly immunodeficient mice must be used as host. Especially mice with the NOD/shi-scid IL-2rγ(-/-) (NSG) background as well as the humanized version have proved to be reliable for the evaluation of efficacy and safety of CAR-T therapies. Due to the high immunodeficient state of the strains, these models are not, however, suited to investigate the complex interactions with other immune cell compartments influencing the outcome of the therapy in the context of lymphodepletion.

Therefore, a variety of syngeneic murine models has been developed to study the efficacy of adoptively transferred cells in the presence of the lymphoid compartment. Several decades ago, it was observed that the pre-conditioning with chemotherapeutic agents or total body irradiation has a significant impact on engraftment and therapy outcome (72, 116–118). This experimental evidence has been translated into the application of lymphopenia inducing conditioning as standard procedure to improve the engraftment and persistence of adoptively transferred cells in the clinic (35).

Typically, lymphodepletion in murine model systems is achieved by non-myeloablative total body irradiation (TBI). The irradiation dose to induce the desired pre-conditioning state before adoptive cell transfer can vary depending on the strain of mice and experimental requirements. Gattinoni et al. elucidated the effect of 5 Gy TBI in the context of T-cell Receptor (TCR) transgenic CD8^+^ T cells (pmel-1) targeting the self/tumor antigen gp100 in mice bearing established B16 melanoma. While the pre-conditioning with this intensity induced severe lymphopenia in treated mice, the anti-tumor efficacy was significantly increased compared to non-conditioned mice (77). Similar results were observed in pre-clinical settings with anti-murine CD19 CAR-T. Conditioning with 5-6 Gy provided optimal support for the key factors of efficacy like engraftment, proliferation and tumor eradication in mice challenged with 38c13 or A20 lymphoma and treated with CAR-T (119, 120). Therefore, the common irradiation doses in preclinical models for adoptive cell therapy, also applied in further tumor models, are usually based on a single-dose treatment between 4 to 6 Gray (121, 122).

Besides irradiation, clinically relevant chemotherapeutic agents such as cyclophosphamide and fludarabine as well as combination therapies are used in pre-clinical models to induce the conditioned state for adoptive transfer of T-cells. In a syngeneic C57Bl/6 mouse model of B-ALL Davila et al. applied escalating doses of cyclophosphamide and CAR-T doses (123). While the cyclophosphamide administration in moderate doses of 100 mg/kg improved the survival of treated mice and lead to a reduction in the B cell compartment, a dose escalation amplified the effect even further on the B cell level irrespective of the CAR-T cell dose administered. These results illustrate the central role of intensive pre-conditioning, rather than CAR-T dose escalation. This and other models apply typical cyclophosphamide conditioning from 50 mg/kg to 300 mg/kg in a single-dose treatment (123–126). Selected fludarabine doses are considerably lower ranging from 25 mg/kg to 100 mg/kg (77, 127, 128). Furthermore, not only individual therapy options are considered, Flu/Cy pre-treatments in pre-clinical models are also combined compared to the clinical regimens [16]. While a single-dose cyclophosphamide as well as Flu/Cy regimens induce strong leukopenia in mice, a single-dose fludarabine alone does not affect the leukocyte compartment (129).

While these studies demonstrate the importance of conditioning for engraftment and persistence of tumor reactive T cells in pre-clinical models, the effects of pre-conditioning on recipient mice that generate the most favorable milieu for cell transfer are complex and diverse.

In general, the combination of different mechanisms might be responsible for the advantage in engraftment and persistence of administered T cells in conditioned recipients rather than one mechanism alone.

Homeostatic expansion of T-cells has been one of the proposed mechanisms that drive anti-tumor immunity (86)(see also chapter influence of tumor microenvironment). The size of the immune compartment is precisely controlled through homeostatic regulation of the level and activation state of each individual cell type. Pre-conditioning induced lymphopenia triggers the expansion of T-cells by recognition of self-MHC/peptide ligands to restore the initial T-cell compartment size. In this process, naïve T-cells differentiate into activated/effector T-cells and gain effector functions characterized by interferon production. Surprisingly, in the pmel-1 transgenic mouse model challenged with B16 melanoma, the irradiation induced an increase in the functional capacity rather than the specific expansion of anti-tumor T-cells. While the compartment size of transferred T-cells was comparable between conditioned and non-conditioned mice, the interferon, tumor necrosis factor and IL-2 production were increased in irradiated mice (77). These results indicate a conditioning induced shift towards a reduced activation threshold of transferred T-cells.

In line with these findings, lymphodepletion has shown to reduce the levels of immune suppressive cells such as CD4^+^/CD25 ^+^/FoxP3 ^+^ Treg and myeloid derived suppressor cells that contribute to immune tolerance by maintaining the activation threshold of effector T-cells (130). These cells maintain immunological tolerance to self/tumor antigens (131, 132) (see also chapter microenvironment).

In the pmel-1 mouse model, the adoptive transfer of CD4 T helper cells depleted by the regulatory CD25 type improved the anti-tumor response, while the transfer including the regulatory compartment significantly decreased anti-tumor efficacy indicating the critical role of lymphopenia inducing conditioning (133). Moreover, especially low dose cyclophosphamide is reported to selectively deplete Treg with a less suppressive capacity after recovery (126, 134, 135). Interestingly, it could be shown in a RAG^-/-^ mice, which are naturally deficient in Treg cells, that non-myeloablative irradiation potentiates the efficacy of the treatment with tumor-reactive lymphocytes (77). Antibody mediated depletion of NK cells abolished this effect leading to similar tumor-control as in conditioned mice. Transferring the cells into tumor-bearing mice deficient in the homeostatic cytokines IL-7 or IL-15 impairs the treatment effect irrespective of conditioning. Based on these results, it was postulated, that lymphodepletion removes cellular compartments, that act as cytokine sinks and increases the cytokine availability of tumor-reactive transferred T cells (77).

While lymphodepletion with irradiation and Flu/Cy treatment significantly enhances adoptive cell therapy with tumor reactive lymphocytes in murine und human settings, the occurrence of life-threatening toxicities associated with pre-conditioning strategies demonstrates the need to overcome this hurdle.

Even though the transfer of *in vitro* activated and engineered T-cells in non-conditioned recipients remains challenging, engraftment of extremely low T-cell numbers, as low as a single naïve T-cell, can already be sufficient to develop into highly effective effector and memory T-cell subsets in non-lymphodepleted hosts (136). Several studies also investigated the potential of different phenotypic T-cell compositions on the potency to induce tumor destruction, a strategy that might also impact the dependence on the engraftment and persistence potential in lymphoreplete mouse models. Especially T-cell subsets of the stem cell memory (TSCM) and central memory (TCM) compartment possess superior functionality and persistence potential compared to more differentiated subsets (137–139). Therefore, ultra-short manufacturing protocols and the transfer of highly defined T cell subsets might be the strategy to preserve long-term persistence, while minimizing or preventing pre-conditioning requirements.

The co-engineering of T-cells to express tumor specific receptors in combination with interleukins is another promising strategy to overcome the conditioning obstacle. For example, a second generation anti-murine CD19 CAR construct co-expressing IL-12 was reported to successfully eliminate A20 lymphoma cells and to improve survival in lymphodepleted mice (125). Proposed mechanisms by which IL-12 co-expression leads to a functionality improvement even in the absence of pre-conditioning are the induction of proinflammatory cytokines, activation of NK and DC cells as well as an improvement in the cytotoxic effector function of T cells (140–142). Similar results were generated by the co-expression of the anti-murine CD19 CAR with IL-18 (143). As recombinant or transgenic cytokine delivery improves functionality, it comes at the cost of significant toxicities limiting clinical application. Therefore, the selective constitutive stimulation via engineered interleukin receptors like the IL-7 receptor or IL-2 incorporated in the CAR transgene as well as vaccination strategies provide a safer more promising approach for clinical translation (144–146). This co-engineering might also be a valuable approach to overcome CAR-T cell application in the solid tumor context moving more and more into the focus of the field (147). In summary murine model systems are essential in the toolbox for designing novel CAR-T products with reduced or even omitted LD requirements.

## Impact of LD on CAR-T cellular kinetics in humans

Factors that impact toxicity and efficacy have been difficult to define because of differences in lymphodepletion regimens and heterogeneity of CAR-T administered to individuals. There have been many studies that investigated the effects of higher intensity vs. lower intensity of lymphodepletions on CD19 CAR-T expansion and persistence and clinical responses (**Figure 2B**). Patients, who received Flu/Cy lymphodepletion, had markedly increased CAR-T expansion and persistence (109). In addition, Curran et al., 2019 showed that higher dose intensity of conditioning chemotherapy and minimal pretreatment disease burden have a positive impact on response without a negative effect on toxicity (148). Higher intensity lymphodepletion clearly improves the cell expansion and persistence along with better efficacy rates; it is, however, usually associated with higher toxicity rates. Flu 25 mg/m2 x 2 + Cy 250 mg/m2 X 3 or Benda 90 mg/m2 x 2 (tisa-cel, DLBCL/FL) was also used in LBCL with tisa-cel as LD agent and results showed that Benda is as effective as Flu/Cy and validates a safer adverse event profile with reduced CRS, Immune Effector Cell-Associated Neurotoxicity Syndrome (ICANS), and hematological toxicities (53). These chemotherapeutic drugs can, however, increase the risk of infections and other side effects leading to the development of secondary malignancies.

## Clinical effects

### Impact on safety

It rapidly became clear that LD chemotherapy was not toxicity-free (149), as an early report describes how a patient with R/R CLL developed an unexpected toxic death (hypotension, dyspnea and renal failure) after adding LD with 1.5 g/m2 Cy before administering a 19-28z CAR-T. This was the first patient on this trial receiving CAR-T after LD, as the preceding 3 patients were safely treated without LD but with lack of CAR-T engraftment or efficacy. This experience made clear that LD might not only increase desired adoptive cell functions, but also their related toxicities. Later, further patients were treated in this trial (150).Almost no expansion and no clinical responses were seen among patients that received treatment without prior LD (0/3), in contrast to the 4/9 patients that did receive cyclophosphamide as LD that achieved better engraftment.

Almost at the same time, another case report (18)on a R/R CLL patient presented a patient treated with another second generation anti-CD19 (FMC63), 4-1BB-CD3z-based autologous CAR-T. The patient received prior LD with 600mg/m2 cyclophosphamide plus pentostatin 4mg/m2. These drugs were chosen due to their potential double effect (LD plus antitumor activity) as they already were being used to treat CLL (151). Surprisingly, the administration of what was considered a very low CAR-T dose (1.46×10e5 CAR-T cells/kg) led to an unprecedented MRD-negative response and deep B-cell aplasia. Similar outcomes were seen with 2 subsequent patients treated on the same trial that received LD with other agents (benda alone or with rituximab) (152).

Cytopenias and subsequent infections are one of the most important long-term side effects. Neutropenia and lymphopenia both contribute to the development of gram-negative or other opportunistic infections (153) Interestingly, a biphasic curve is observed for PB cell counts. The initial drop in white blood cells (within the first 2 weeks) is probably related to LD therapy (154). We have recently obtained evidence that not only mature myeloid or lymphatic cells are decreased during this phase, but that peripheral blood hematopoietic progenitors are also diminished (155) (**Figure 2A**). This indicates the marked toxicity of Flu/Cy - or benda-based regimens on all maturation grades of hematopoietic cells. Furthermore, it is unclear whether fludarabine levels remaining after CAR-T cell infusion (e.g. in patients with reduced kidney function) have detrimental effects on the number and function of CAR-T cells (61, 62). Recent evidence suggests that benda might be associated with fewer infections (53, 156).

While not being the main factor, LD is also likely to affect other known toxicities of CAR-T therapy including CRS and neurotoxicity. LD with Flu/Cy was identified as an independent variable of baseline characteristics together with high marrow tumor burden, higher CAR T-cell dose, thrombocytopenia before lymphodepletion, and manufacturing of CAR-T without selection of CD8^+^ central memory T cells as independent predictors of CRS (157).

The direct influence of LD on neurotoxicity is unclear, but parameters obtained at the start of LD predict severity of toxicity (158).

Due to the broad toxicity of Flu, Cy or benda, effects on other body systems such as the intestinal mucosa and the microbiome are also expected (159). This could facilitate the development of early gram-negative infections during neutropenia.

### Impact on response to CAR-T cells

A direct influence of LD intensity on clinical response rates is well documented (see above). However, the strict connection between certain LD regimens and approved CAR-T products make a meta-analysis based on large datasets virtually impossible. However, it is interesting to note that in aggressive lymphoma the two products with higher Flu/Cy doses (axi-cel and liso-cel) have higher response rates and better long-term survival parameters then tisa-cel (63) Through their influence on cellular kinetics, LD regimens could also be involved in modulation of response.

## Best current approaches

The best current approach may vary depending on indication and product. However, it seems that Flu/Cy at doses of 90mg/900-1500mg is a reasonable choice for most autologous settings. The data suggesting an optimal therapeutic window may lead to individualized dosing, particularly for fludarabine (115). Recent evidence suggests that bendamustin (180mg) is equally effective and possibly less toxic with both, tisa-cel and axi-cel in lymphoma and with cita-cel and ide-cel in multiple myeloma (53, 160, 161). Moreover, Benda is the preferred LD for patients with renal insufficiency or other comorbidities. For allogeneic CAR-T cells the evidence is less clear. Alemtuzumab based LD is a preferred option in the allogeneic setting, but has considerable toxicity.

## Open questions and future developments

While LD is firmly established in the routine CAR-T cell treatment cycle, it seems clear that improvements in this area are an unmet need. We note the paucity of systematic investigations of agents, dosage and combinations (animal and human data). This includes the lack of comparability between trials and real-world evidence data due to variability and close connection of LD to (approved) therapies. Further investigation of individual metabolism including comorbidities and ethnic differences will help to personalize LD. Optimal LD in solid tumors or non-malignant diseases has yet to be established. Finally, recent studies exploring ultra-short manufacturing protocols or *in vivo* gene editing indicate that with the advent of less *in vitro* cell culture-dependent strategies and technologies, the dependency of CAR-T engraftment, expansion and survival on LD is strongly reduced and could in certain situations even be omitted (162, 163). Furthermore, the co-engineering strategies with receptors or the implementation of vaccination strategies to selectively boost the proliferation and expansion of adoptively transferred T-cells represents another exciting approach to overcome hurdles associated with LD (144–146).

## Conclusions

Lymphodepletion is essential for the success of currently used CAR-T therapies. Detailed laboratory investigations on drugs, dosage and timing are, however, still needed for informed selection of regimens and novel drugs. Clinical data on the specific effects of LD on cellular kinetics, efficacy as well as toxicity should be obtained through real world experience as well as dose finding studies. The T2EVOLVE consortium and others are currently addressing many of these question in a concerted action to pave the way for standardized and optimized designs of LD regimens, aiming to improve patient care and therapy success in the future.

## Author contributions

BL: Writing – original draft, Writing – review & editing. LC: Writing – original draft, Writing – review & editing. VO-M: Writing – original draft, Writing – review & editing. LW: Writing – original draft, Writing – review & editing. PB: Writing – review & editing. MD: Writing – review & editing. DH: Writing – review & editing, Writing – original draft. MH: Writing – review & editing. AK: Writing – review & editing. JM: Writing – review & editing. MN: Writing – review & editing. HN: Writing – review & editing. CS: Writing – review & editing, Writing – original draft. PS: Writing – review & editing. RT: Writing – review & editing. DB: Writing – review & editing. JD: Writing – review & editing. JK: Writing – original draft, Writing – review & editing. ML: Writing – original draft, Writing – review & editing. UJ: Writing – original draft, Writing – review & editing.
